# Association of serum uric acid with anemia in U.S. adults: a cross-sectional study using secondary data

**DOI:** 10.1186/s12872-023-03291-y

**Published:** 2023-06-08

**Authors:** Yingying Wang, Jingjing Ming, Zongyun Guo, Wei Zhang, Xiang Li, Shengguo Zhou, Xia Li, Huijuan Ma

**Affiliations:** 1 Department of Nephrology, Jining No.1 People’s Hospital, Jining, Shandong China; 2 Department of Emergency Trauma Surgery, Jining No.1 People’s Hospital, Jining, Shandong China; 3 Department of Nephrology, Affiliated Hospital of Jining Medical University, No.6 Jiankang Road, Jining, 272011 Shandong China

**Keywords:** anemia, Serum uric acid, U-shaped curve, US adults

## Abstract

**Background:**

High serum uric acid (SUA) is a risk factor of cardiovascular disease (CVD). Abnormal SUA have been correlated with a significant increase in mortality. Anemia is an independent predictor of mortality and CVD. To date, no study has investigated the relationship between SUA and anemia. Here, we explored the correlation between SUA and anemia in the American population.

**Methods:**

The cross-sectional study involved 9205 US adults from NHANES (2011–2014). The relationship between SUA and anemia was explored using multivariate linear regression models. Two-piecewise linear regression model, generalized additive models (GAM) and smooth curve fitting were performed to explore the non-linear relationships between SUA and anemia.

**Results:**

We found a U-shaped non-linear relationship between SUA and anemia. The inflection point of the SUA concentration curve was 6.2 mg/dL. The ORs (95% CIs) for anemia on the left and right of the inflection point were 0.86 (0.78–0.95) and 1.33 (1.16–1.52), respectively. The 95% CI of inflection point was 5.9-6.5 mg/dL. The findings showed that both genders presented a U-shaped correlation. Safe ranges of SUA in men and women were 6-6.5 and 4.3–4.6 mg/dL, respectively.

**Conclusions:**

Both high and low SUA levels were correlated with increased risk of anemia, and a U-shaped relationship was observed between SUA and anemia.

**Supplementary Information:**

The online version contains supplementary material available at 10.1186/s12872-023-03291-y.

## Background

Uric acid (UA) is formed during nucleic acids metabolism through a reaction catalyzed by xanthine oxidase [1]. Serum uric acid (SUA) is elevated by increased production and/or decreased excretion of uric acid. Elevated SUA has negative effects on health and is associated with estimated glomerular filtration rate (eGFR) decline and chronic kidney disease(CKD)progression, gout, increased mortality, cardiovascular disease (CVD), hypertension, metabolic syndrome [2–8].

Low SUA levels can increase the risk of CVD, and a J-shaped correlation has been reported between SUA levels and cardiovascular risk [9]. Moreover, high and low SUA levels are linked to a significant increase in cause-specific and all-cause mortality, indicating a U-shaped relationship between SUA and mortality [10].

Anemia is caused by low hemoglobin (Hb) level and is characterized by reduced blood oxygen-carrying capacity of red blood cells [11]. Anemia is an independent predictor for mortality and CVD [12]. In 2010, the global prevalence of anemia was 32.9% [13]. Thus, exploring the risk factors of anemia may reveal strategies for anemia prevention and management of adverse outcomes.

To date, few studies have explored the association between anemia and SUA. A study on the association between gout and anemia indicated that anemia is an independent risk factor for gout in middle-aged people. In addition, the findings showed that anemia was associated with approximately 2 times increased risk of gout, independent of kidney function and SUA [14]. A study by the Korean National Health and Nutrition Examination reported that anemia was associated with increased prevalence of CKD and the risk of hyperuricemia in CKD subjects was 2-fold higher compared with the risk in health individuals, after adjusting for renal function [15]. In summary, anemia is associated with increased risk of hyperuricemia or gout.

SUA is positively correlated with Hb levels. A previous study in a Chinese Adult Population showed that Hb in the hyperuricemia group was higher than that in the control group [16]. The SUA level was significantly positively correlated with Hb levels in a retrospective study of 607 adolescents [17]. However, to our knowledge, the relationship between SUA and anemia in the general population has not been clarified. The current study explored the correlation between SUA and anemia by analyzing data retrieved from the National Health and Nutrition Examination Survey (NHANES), which is representative of the American population.

## Methods

### Study population and design

National Health and Nutrition Examination Survey (NHANES) is a cross-sectional survey conducted every two years. NHANES is used by the Centers for Disease Control and Prevention (CDC) to analyze the nutritional and health status of people in the U.S [18]. It uses multi-staged, stratified, sampling design to select representative U.S. participants to be interviewed and undergo health checkup at a mobile examination center (MEC) [19].

Two surveys (2011–2012 and 2013–2014) involving 19,931 participants and containing information on SUA and anemia, were included in the current analysis. A total of 11,329 participants were ≥ 20 years old. After excluding cases with missing data on SUA (n = 1116) and hemoglobin (n = 8), cases with cancer (n = 896), cases with extreme SUA>11 mg/dl (n = 9), and cases treated with allopurinol during the month prior to the survey (n = 95), 9205 participants were finally enrolled in the current study.

#### Ethical approval

The study was approved by the ethics review committee of the National Center for Health Statistics. All participants provided written informed consent.

### Variables

SUA was the exposure variable and was measured by DxC800 using a timed endpoint method. The outcome variable was the presence or absence of anemia. Based on the World Health Organization (WHO) criteria [20], anemia was defined as Hb < 12 g/dL and < 13 g/dL in women and men, respectively. Hb was measured on a Beckman Coulter MAXM instrument. Hyperuricemia was defined as serum uric acid level ≥ 7.0 mg/dL in men and ≥ 6.0 mg/dL in women. The following potential confounding factors were included in the analysis as covariates: poverty-income ratio (< 1 and ≥ 1), academic level (below high school, high school, and above high school), age, smoking status (never smoker, former smoker and current smoker), gender, race (Non-Hispanic Black, Non-Hispanic White, other Hispanic and other races and Mexican American), marital status (never married, living with partner/married and divorced/widowed/separated), body mass index (BMI), drinking (intake of a minimum of 12 alcohol drinks annually or not), total cholesterol, red blood cell folic acid, vitamin B12, albumin, serum iron, white blood cells, platelets, urinary albumin-to-creatinine ratio (UACR) and eGFR. The diagnosis of CKD was as follows: eGFR = < 60mL/min/1.73m^2^. eGFR was calculated using a CKD epidemiology collaboration formula as shown: [21] GFR = 141 × 1.159 [if black], × max (Scr/K,1)-1.209 × min (Scr/K,1)a × 1.018 [if female] × 0.993^Age^, where Scr indicates plasma creatinine (mg/dL), K is constant whereby the value is 0.9 for males and 0.7 for females, a is constant whereby the value is -0.411 for males and − 0.329 for females, max represents the highest Scr/K level or 1 and min represents the lowest Scr/K level or 1. History of diabetes, hypertension, heart failure or coronary heart disease were indicators of self-reported physician diagnoses. Details on covariates, SUA and Hb levels are available in the CDC database (http://www.cdc.gov/nchs/nhanes/).

### Statistical analysis

Continuous variables with normal distribution were presented as mean ± SD. Data with skewed distribution were presented as medians (quartiles). Categorical variables were presented as frequencies or percentages. Statistical significance was calculated using one-way ANOVA for normally distributed data. Kruskal-Wallis H test was used to compare data with skewed distribution. Differences between categorical variables were compared by the chi-square test. SUA was a continuous variable, thus the smooth curve fitting and generalized additive models (GAM) were used to explore non-linear relationships. For non-linear correlations, a two-piecewise linear regression model was established to calculate the relationship between SUA and anemia using a smoothing plot. Unadjusted, minimally adjusted, and fully adjusted findings were presented in line with the STROBE guidelines [22]. Subgroup analyses were performed using stratified linear regression models. Modifications and interactions among subgroups were examined through likelihood ration tests. All statistical analyses were performed in R software (http://www.R-project.org, The R Foundation, version 4.2.0) and EmpowerStats (http://www.empowerstats.com, X&Y Solutions, Inc., Boston, MA). Values with *P* = < 0.05 (two-sided) were considered statistically significant.

## Results

### Baseline characteristics of participants

A total of 9205 participants (mean age: 47.15 ± 17.09 years, 48.55% males) aged ≥ 20 years, met the inclusion criteria. Participant features were subclassified based on the presence or absence of anemia (non-anemia and anemia, Table 1). The results showed that characteristics of non-anemia participants were significantly different from those with anemia. Relative to the non-anemia group, there were more black patients, female subjects, low education level, low total levels of cholesterol, eGFR, albumin, serum iron and SUA, higher age, UACR and BMI in the anemia group. Those with hypertension, diabetes, hyperuricemia and heart failure were more likely to be anemic.

**Table 1:** Baseline Characteristics of Participants

characteristics	Non-anemia	Anemia	*P*-value
N	8197	1008	
Age (years)	46.60 ± 16.88	51.61 ± 18.10	< 0.001
gender (n, %)			< 0.001
male	4151 (50.64%)	318 (31.55%)	
female	4046 (49.36%)	690 (68.45%)	
Race (n, %)			< 0.001
mexican American	1036 (12.64%)	114 (11.31%)	
non-Hispanic white	3307(40.34%)	201 (19.94%)	
non-Hispanic black	1662 (20.28%)	467 (46.33%)	
other Hispanic	826 (10.08%)	78 (7.74%)	
other races	1366 (16.66%)	148 (14.68%)	
Academic level (n, %)			< 0.001
below high school	1789 (21.84%)	275 (27.34%)	
high school	1787 (21.81%)	222 (22.07%)	
above high school	4616 (56.35%)	509 (50.60%)	
Marital status (n, %)			< 0.001
living with partner/married	4816 (58.77%)	516 (51.24%)	
divorced/widowed/separated	1626 (19.84%)	271 (26.91%)	
never married	1753 (21.39%)	220 (21.85%)	
Poverty-income ratio (n, %)			< 0.001
< 1	1789 (23.68%)	267 (29.15%)	
>=1	5767 (76.32%)	649 (70.85%)	
Smoking status (n, %)			< 0.001
never smoker	4663 (56.94%)	676 (67.13%)	
former smoker	1766 (21.56%)	201 (19.96%)	
current smoker	1761 (21.50%)	130 (12.91%)	
Drinking (n, %)			< 0.001
no	1921 (25.86%)	335 (37.60%)	
yes	5507 (74.14%)	556 (62.40%)	
Hypertension (n, %)			< 0.001
no	5532 (67.56%)	555 (55.11%)	
yes	2656 (32.44%)	452 (44.89%)	
Diabetes (n, %)			< 0.001
no	7345 (89.67%)	795 (78.87%)	
yes	846 (10.33%)	213 (21.13%)	
congestive heart failure (n, %)			< 0.001
no	8005 (97.81%)	939 (93.34%)	
yes	179 (2.19%)	67 (6.66%)	
coronary heart disease (n, %) no yes Hyperuricemia(n, %) no yes UACR(mg/g) White blood cells (10^3^ cells/ul ) Platelets (10^3^ cells/ul) Serum iron (ug/dl)	7931 (97.02%) 244 (2.98%) 6658(81.22%) 1539 (18.78%) 6.82(4.50-12.64) 6.90(5.70–8.40) 234.90 ± 57.39 83.00(63.00-106.00)	951 (94.72%) 53 (5.28%) 779 (77.28%) 229 (22.72%) 8.70(5.49–22.78) 6.60(5.30–8.20) 252.38 ± 75.82 55.00(34.00–78.00)	< 0.001 0.003 < 0.001 < 0.001 < 0.001 < 0.001
Vitamin B12(pg/ml)	383.00(284.90-525.50)	397.00(276.00-569.00)	0.100
RBC folate (ng/mL)	459.00(351.00-600.00)	472.00(347.00-662.00)	0.015
Total cholesterol(mg/dl)	192.72 ± 40.82	178.61 ± 41.35	< 0.001
eGFR(mL/min/1.73m^2^)	97.87 ± 23.43	91.96 ± 33.62	0.001
Uric acid (mg/dl,)	5.43 ± 1.38	5.28 ± 1.66	< 0.001
Albumin(g/dl)	4.30 ± 0.32	4.01 ± 0.37	< 0.001
BMI (kg/m^2^)	28.83 ± 6.89	29.76 ± 7.61	0.001

Abbreviations: RBC: red blood cells, BMI: body mass index, eGFR: estimated glomerular filtration rate, UACR: urinary albumin-to-creatinine ratio

### Univariate analysis

The results of univariate analysis are shown in Additional File Table 1. Notably, age, female and BMI were positively correlated with anemia, whereas total cholesterol, eGFR, albumin, serum iron, white blood cells and SUA were negatively associated with anemia. Smokers and alcohol users had a lower incidence of anemia. Black race, hypertension, diabetes or heart failure had higher risks of anemia.

### Association of SUA with anemia

Correlations between SUA and anemia were explored using multivariate linear regression models as shown in Table 2. Three models were constructed to explore the role of SUA in anemia. In the crude model, the odds ratios (ORs) for anemia were 1.05(95%CI:1.00,1.10; *P* = 0.0597), 1.19(95%CI:1.09,1.30; *P*<0.0001) and 0.98 (95%CI:0.92,1.05; *P* = 0.5853) among all population, male and female, respectively. The findings from the minimally adjusted model (model 1) showed the ORs for anemia were 0.97(95%CI:0.92,1.03; *P* = 0.3009), 1.13(95%CI:1.03,1.24; P = 0.0110) and 0.96 (95%CI:0.89,1.03; *P* = 0.2649) among all participants, male and female, respectively. After adjusting for other confounders, we did not detect any connection in a fully adjusted model (model 2) (OR = 1.01, 95% CI: 0.94–1.09; *P* = 0.7555) for the whole people. There were also no statistically significance in men and women. To perform sensitivity analysis, we converted SUA from a continuous variable to a categorical variable (quartiles). When using the lowest quartile of SUA as the reference in all people, multivariable ORs for anemia decreased, but not in parallel with the quartiles of SUA, (ORs were 0.76(95%CI: 0.60, 0.95), 0.75 (95%CI: 0.58, 0.96), and 0.91 (95%CI: 0.70, 1.20)) from the second to the fourth quartiles, respectively. These results suggest that the association between SUA and anemia is likely to be nonlinear.

**Table 2:** Relationship between SUA and anemia in different models

Exposure	Gender = Male	Gender = Female	Total
Crude model			
Uric acid(mg/dl)	1.19 (1.09, 1.30) < 0.0001	0.98 (0.92, 1.05) 0.5853	1.05 (1.00, 1.10) 0.0597
SUA quartiles			
Q1	1.0	1.0	1.0
Q2	0.73 (0.46, 1.15) 0.1720	0.68 (0.56, 0.84) 0.0003	0.71 (0.59, 0.85) 0.0002
Q3	0.62 (0.40, 0.94) 0.0264	0.81 (0.65, 1.02) 0.0676	0.75 (0.62, 0.91) 0.0035
Q4	0.93 (0.62, 1.38) 0.7117	1.09 (0.85, 1.40) 0.4767	1.05 (0.86, 1.27) 0.6321
P for trend	1.05 (0.93, 1.18) 0.4528	0.99 (0.92, 1.07) 0.8235	1.01 (0.94, 1.08) 0.8209
Model 1			
Uric acid(mg/dl)	1.13 (1.03, 1.24) 0.0110	0.96 (0.89, 1.03) 0.2649	0.97 (0.92, 1.03) 0.3009
SUA quartiles			
Q1	1.0	1.0	1.0
Q2	0.90 (0.54, 1.49) 0.6779	0.68 (0.54, 0.85) 0.0009	0.65 (0.53, 0.80) < 0.0001
Q3	0.70 (0.43, 1.14) 0.1516	0.73 (0.57, 0.95) 0.0178	0.60 (0.48, 0.75) < 0.0001
Q4	0.98 (0.62, 1.55) 0.9298	1.01 (0.76, 1.35) 0.9410	0.78 (0.63, 0.97) 0.0250
P for trend	1.02 (0.90, 1.17) 0.7436	0.96 (0.87, 1.05) 0.3586	0.91 (0.85, 0.98) 0.0141
Model 2			
Uric acid(mg/dl)	1.12 (1.00, 1.25) 0.0578	1.03 (0.93, 1.13) 0.5971	1.01 (0.94, 1.09) 0.7555
SUA quartiles			
Q1	1.0	1.0	1.0
Q2	0.97 (0.56, 1.67) 0.9003	0.77 (0.59, 1.00) 0.0534	0.76 (0.60, 0.95) 0.0183
Q3	0.78 (0.46, 1.33) 0.3692	0.92 (0.68, 1.25) 0.6154	0.75 (0.58, 0.96) 0.0215
Q4	1.04 (0.62, 1.75) 0.8795	1.24 (0.85, 1.79) 0.2643	0.91 (0.70, 1.20) 0.5193
P for trend	1.02 (0.87, 1.19) 0.7845	1.04 (0.92, 1.17) 0.5249	0.96 (0.88, 1.05) 0.4195

Crude model: we did not adjust for other covariants

Model 1: adjusted for age, gender, race, academic level, marital status, smoking status, drinking

Model 2: adjusted for age, gender, race, academic level, marital status, BMI, smoking status, drinking, RBC folate, Vitamin B12, total cholesterol, hypertension, diabetes mellitus, congestive heart failure, coronary heart disease, eGFR, white blood cells, platelets, albumin, serum iron

### Non-linear association of SUA with anemia

**Fig. 1:**
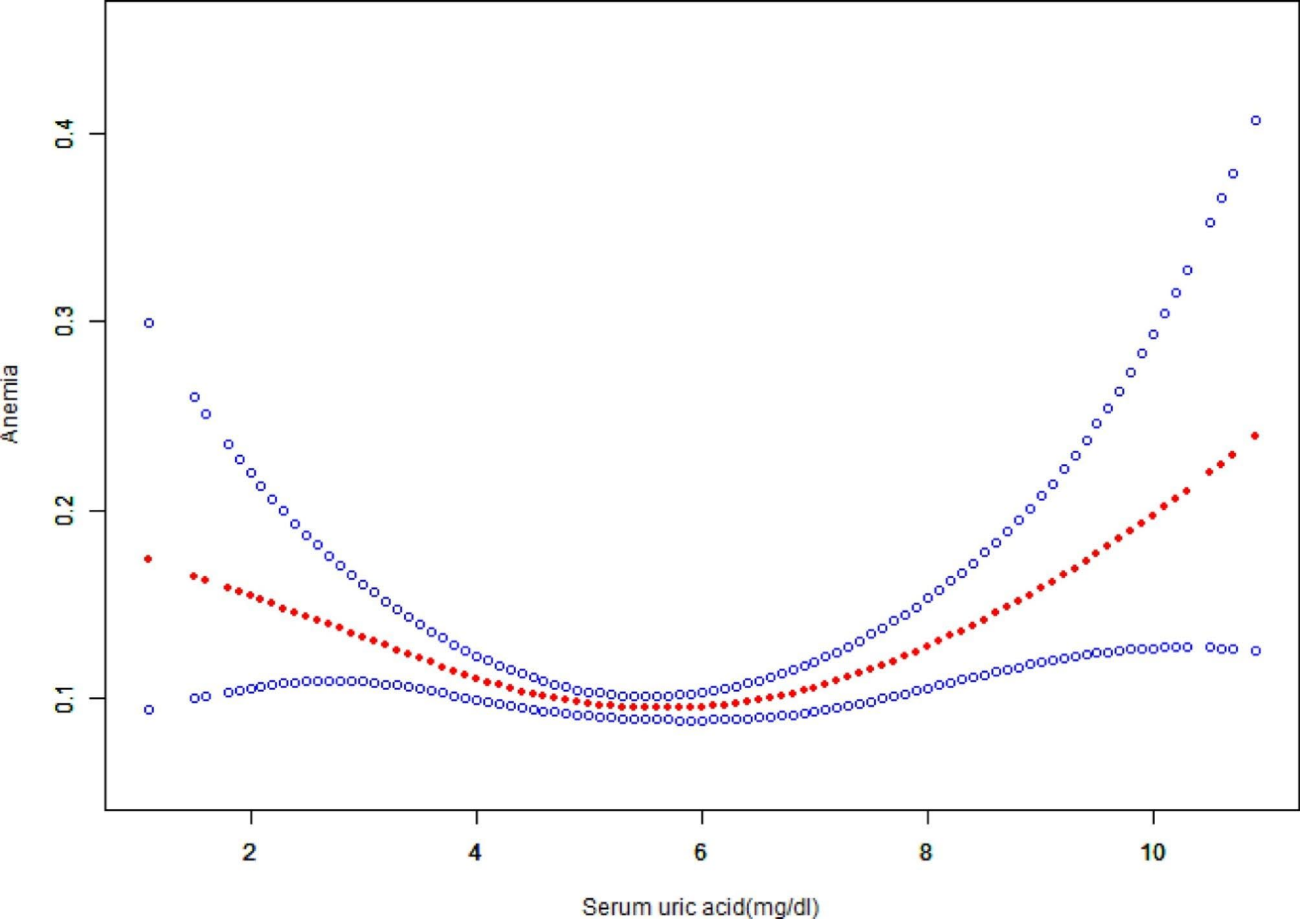
The non-linear relationship between SUA and anemia. Threshold, non-linear relationship between SUA and anemia was found in a generalized additive model (GAM) after adjusting for age, gender, race, academic level, marital status, BMI, smoking status, drinking, RBC folate, Vitamin B12, total cholesterol, hypertension, diabetes mellitus, congestive heart failure, coronary heart disease, eGFR, white blood cells, platelets, albumin, serum iron

Next, we analyzed the non-linear relationship between SUA with anemia and found that the smooth curve fitting between SUA and anemia was U-shaped (Fig. 1) after full adjustment for marital status, educational level, gender, age, smoking status, alcohol use, race, BMI, hypertension, diabetes mellitus, congestive heart failure, coronary heart disease, vitamin B12, RBC folate, total cholesterol, white blood cells, platelets, albumin, serum iron, and eGFR.

A threshold effect analysis was further conducted. The inflection point of SUA was at 6.2 mg/dL as indicated by a two-piece wise linear regression model. The log likelihood ratio test showed that the value of *P* was < 0.001. The indicated that elevated SUA was negatively correlated with reduced risk of anemia (0.86, 0.78 to 0.95, 0.0029) on the left of the inflection point (≤ 6.2 mg/dL). Conversely, the analysis showed that elevated SUA was significantly positively correlated with high risk of anemia (1.33, 1.16 to 1.52, < 0.0001, Table 3) on the right side of the inflection point (> 6.2 mg/dL). Based on the 95% CI of inflection point (5.9-6.5 mg/dl), the rate of anemia was lowest in the overall population.

**Table 3:** Threshold Effect Analysis for SUA and anemia using Piece-wise Linear Effect: anemia cause: SUA

Gender	Male	Female	Total
Inflection point of uric acid(mg/dl)	6.3	4.4	6.2
On the left	0.89 (0.73, 1.08) 0.2406	0.72 (0.58, 0.91) 0.0047	0.86 (0.78, 0.95) 0.0029
On the right	1.40 (1.15, 1.70) 0.0008	1.18 (1.04, 1.33) 0.0096	1.33(1.16, 1.52) < 0.0001
P for log likelihood ratio test	0.007	< 0.001	< 0.001
95% CI of inflection point	6, 6.5	4.2, 4.5	5.9, 6.5

Adjusted: age, gender, race, academic level, marital status, BMI, smoking status,

drinking, RBC folate, Vitamin B12, total cholesterol, hypertension, diabetes mellitus, congestive heart failure, coronary heart disease, eGFR, white blood cells, platelets, albumin, serum iron

### Non-linear association of SUA with anemia by gender

**Fig. 2:**
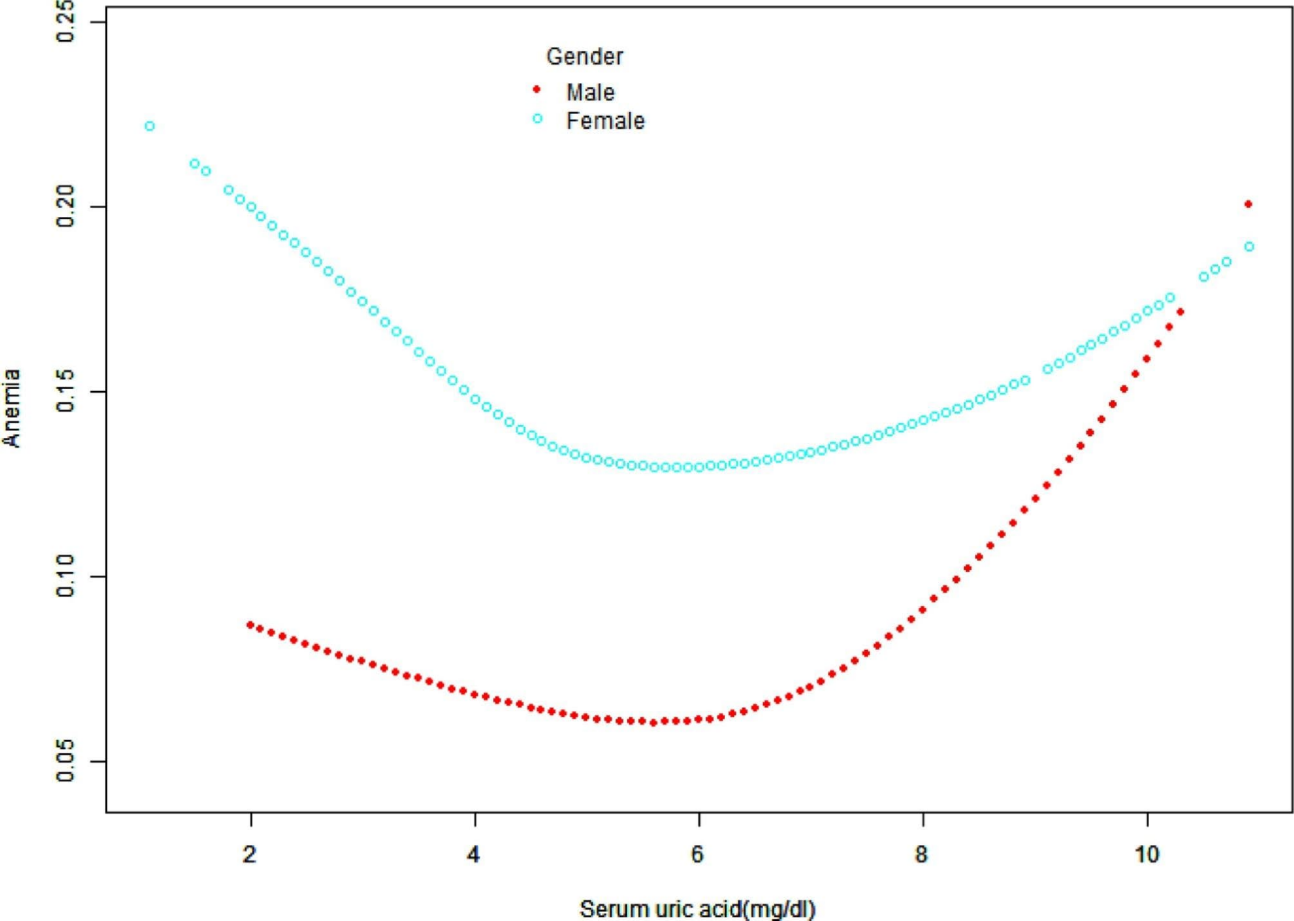
The non-linear relationship between SUA and anemia by sex. SUA and anemia had U-shaped correlation in both sexes after adjusting for age, race, academic level, marital status, BMI, smoking status, drinking, RBC folate, Vitamin B12, total cholesterol, hypertension, diabetes mellitus, congestive heart failure, coronary heart disease, eGFR, white blood cells, platelets, albumin, serum iron

SUA levels differed significantly between men and women. Analysis of the non-linear relationship between SUA and anemia in men and women revealed that SUA and anemia had U-shaped correlation in both sexes (Fig. 2) but SUA inflection point differed in men vs. women (6.3 mg/dL vs. 4.4 mg/dL, Table 3). In men, the ORs were 0.89 (95%CI: 0.73–1.08) when SUA was < 6.3 mg/dL and 1.40(95% CI: 1.15–1.70) when SUA was > 6.3 mg/dL. In women, the ORs were 0.72 (95% CI: 0.58–0.91) when SUA was < 4.4 mg/dL and 1.18 (95%CI: 1.04–1.33) when SUA was > 4.4 mg/dL. The safe SUA range in men and women were 6-6.5 mg/dL and 4.2-4.5 mg/dL, respectively.

### The results of subgroup analyses

Subgroup analyses were performed to determine whether the association between SUA and anemia varies according to participant characteristics. As shown in Additional File Table 2,

SUA was negatively associated with anemia in Mexican American (OR:0.71, 95%CI: 0.54–0.93) and positively associated with anemia in Non-Hispanic black (OR:1.13, 95%CI: 1.01–1.25).

## Discussion

In this study, we explored the correlation between SUA and anemia using a large dataset representing the American population. Our analyses revealed a U-shaped, independent association between SUA and anemia. The findings showed a U-shaped relationship between SUA and anemia stratified by sex. The SUA inflection point determined using two-piecewise regression model was 6.2 mg/dL. SUA threshold values varied by sex. The SUA inflection point was 4.4 mg/dL for females and 6.3 mg/dL for males. The safe range of SUA in men and women was 6-6.5 mg/dL and 4.2-4.5 mg/dL, respectively. The results showed that SUA level was a good predictor of anemia and can be leveraged to develop new hyperuricemia guidelines and update existing one.

Our data revealed a negative correlation between SUA and anemia on the left of the inflection point, which may reflect nutritional status. The metabolic syndrome prevalence increased substantially with the levels of SUA. High obesity incidence, high BMI rate and high energy intake in hyperuricemic participants implied that they had a healthy nutrition compared with non-hyperuricemic subjects [22]. Malnutrition plays an important role in development of anemia [23, 24]. Low SUA levels may coincide with vitamin C deficiency in malnourished people [25]. Vitamin C deficiency, the most effective inducer of iron absorption through non-heme processes, may aggravate anemia since iron deficiency anemia (ID) is the most common type of anemia [26]. Studies have reported that SUA levels are positively correlated with iron level [27]. Thus, low SUA was more likely to occur in anemia caused by iron deficiency within a certain range. Moreover, this relationship remained significant even after adjustment for iron in this study.

Epidemiological evidence show that high UA level is a key risk factor for oxidative stress and is associated with early onset of metabolic, renal and cardiovascular disorders [28]. Intracellular hyperuricemia causes inflammatory responses via activation of cyclooxygenase 2 and release of reactive nitrogen species (RNS) or reactive oxygen species (ROS). A study by Nagababu et al. demonstrated that oxidative stress was increased in iron-deficiency mice using fluorescence measurements. Higher fluorescence intensity was recorded in erythrocyte lysates from iron-deficient mice with anemia that in samples from the control mice [29]. In addition, oxidative stress stimulates cation channels in erythrocytes thereby enhancing clearance of the iron-deficient erythrocytes, resulting in anemia in ID [30].

High level of uric acid was an indicator of acute, chronic and severe inflammation [31, 32]. SUA exhibits pro-inflammatory properties by the activation of the mitogen-activated protein kinase (MAPK) pathway and the phosphatidylinositol-3 kinase (PI3K)- Akt pathway, the inhibition of the adenosine monophosphate-activated protein kinase (AMPK) pathway, and the decline in nitric oxide (NO) synthesis [33]. High SUA levels induce NLRP3 inflammasome and the release of interleukin-1β, ultimately stimulating an inflammatory cascade reaction [34]. In community-dwelling older persons, SUA was positively correlated with IL-1ra, white blood cells, c-reactive protein, IL-18, neutrophil count, IL-6 and TNF-α. Abnormally high levels of c-reactive protein and IL-6 were observed in the SUA quintiles [31]. In chronic inflammatory diseases, anemia is caused by low level of circulating iron. Inflammatory cytokines such as IFN-γ, IL-1 and TFN-α inhibit erythropoiesis and oxidizing agents cause damage to red blood cell membrane which reduces the lifespan of red blood cells [35]. Thus, the positive correlation between high SUA level and anemia on the right of the inflection point may be driven by oxidative stress and inflammatory states.

Both anemia and hyperuricemia are associated with CKD progression. However, a significant association between anemia and uric acid was found even after adjustment for kidney function. This suggests a direct relationship between the two conditions, and not that they are merely coexisting conditions associated with renal function deterioration.

This study had a few limitations. First, the causal relationship was not be determined although it explored the correlation between SUA levels and anemia owing the cross-sectional nature of the study. Second, the current study did not assess the correlation between various types of anemia and uric acid level. Finally, reliance on data from an American population limits the generalizability of our findings. Further studies should determine whether the relationship between SUA and anemia exists in other populations and determine the mechanism underlying the role of SUA in anemia. Notably, the current study was the first to use a smooth curve to discover the non-linear connection between SUA and anemia thus it provides a basis for further studies.

## Conclusions

In conclusion, the findings show that high and low SUA levels are linked to increasing anemia. Our analysis identified a non-linear relationship between SUA and anemia and revealed a U-shaped relationship for the entire population and the two genders. The SUA inflection point was 6.2 mg/dL. The safe range of SUA was 5.9-6.5 mg/dl, at which anemia rate was lowest. The SUA inflection point for males and females was 6.3 mg/dL and 4.4 mg/dL, respectively. The safe range of SUA in men and women was 6-6.5 mg/dL and 4.2-4.5 mg/dL, respectively, at which anemia rate was lowest. The SUA inflection point in the current study is consistent with reported clinical control threshold for gout patients. High vigilance is necessary when SUA value is above or below these thresholds. Further studies should be conducted to elucidate the biological pathways implicated in the relationship between SUA and anemia.

## Electronic supplementary material

Below is the link to the electronic supplementary material.

Additional File 1: The results of univariate analysis

Additional File 2: Effect size of SUA on anemia in prespecified and exploratory subgroups

## Data Availability

The NHANES datasets are available online: https://www.cdc.gov/nchs/nhanes/index.htm.

## List of Abbreviations

SUA: High serum uric acid
CVD: Cardiovascular disease
EGFR: Estimated glomerular filtration rate
CKD: Chronic kidney disease
Hb: Hemoglobin
NHANES: National Health and Nutrition Examination Survey
BMI: Body mass index
ID: Iron deficiency anemia
WHO: World Health Organization
UACR: Urinary albumin-to-creatinine ratio
GAM: Generalized additive models
CI: Confidence interval
OR: Odds ratio
RNS: Reactive nitrogen species
ROS: Reactive oxygen species
