# Supplementary material for: Association of serum uric acid with anemia in U.S. adults: a cross-sectional study using secondary data
Source: BMC Cardiovasc Disord. 2023 Jun 8;23:291. doi: 10.1186/s12872-023-03291-y (PMC10251553; doi:10.1186/s12872-023-03291-y)
Supplement: Supplementary file 1 — Additional File 1: The results of univariate analysis [file 12872_2023_3291_MOESM1_ESM.doc]

**Supplemental table 1 The results of univariate analysis**

| Variable | Statistics | OR(95%CI) | *P*-value |
| --- | --- | --- | --- |
| Age (years) | 47.15 ± 17.09 | 1.02 (1.01, 1.02) | <0.0001 |
| Gender |  |  |  |
| male | 4469 (48.55%) | Refrence |  |
| female | 4736 (51.45%) | 2.23 (1.94, 2.56) | <0.0001 |
| Race |  |  |  |
| mexican American | 1150 (12.49%) | Refrence |  |
| non-Hispanic white | 3508 (38.11%) | 0.55 (0.43, 0.70) | <0.0001 |
| non-Hispanic black | 2129 (23.13%) | 2.55 (2.05, 3.18) | <0.0001 |
| other Hispanic | 904 (9.82%) | 0.86 (0.63, 1.16) | 0.3211 |
| other races | 1514 (16.45%) | 0.98 (0.76, 1.27) | 0.9060 |
| Academic level |  |  |  |
| below high school | 2064 (22.44%) | Refrence |  |
| high school | 2009 (21.84%) | 0.81 (0.67, 0.98) | 0.0269 |
| above high school | 5125 (55.72%) | 0.72 (0.61, 0.84) | <0.0001 |
| Poverty-income ratio |  |  |  |
| <1 | 2056 (24.27%) | Refrence |  |
| >=1 | 6416 (75.73%) | 0.75 (0.65, 0.88) | 0.0003 |
| Marital status |  |  |  |
| living with partner/married | 5332 (57.94%) | Refrence |  |
| divorced/widowed/separated | 1897 (20.62%) | 1.56 (1.33, 1.82) | <0.0001 |
| never married | 1973 (21.44%) | 1.17 (0.99, 1.38) | 0.0635 |
| Smoking status |  |  |  |
| never smoker | 5339 (58.05%) | Refrence |  |
| former smoker | 1967 (21.39%) | 0.79 (0.66, 0.93) | 0.0044 |
| current smoker | 1891 (20.56%) | 0.51 (0.42, 0.62) | <0.0001 |
| Drinking |  |  |  |
| no | 2256 (27.12%) | Refrence |  |
| yes | 6063 (72.88%) | 0.58 (0.50, 0.67) | <0.0001 |
| Hypertension |  |  |  |
| no | 6087 (66.20%) | Refrence |  |
| yes | 3108 (33.80%) | 1.70 (1.49, 1.94) | <0.0001 |
| Diabetes |  |  |  |
| no | 8140 (88.49%) | Refrence |  |
| yes | 1059 (11.51%) | 2.33 (1.97, 2.75) | <0.0001 |
| Congestive heart failure |  |  |  |
| no | 8944 (97.32%) | Refrence |  |
| yes  Coronary heart disease  no  yes | 246 (2.68%)  8882 (96.76%)  297 (3.24%) | 3.19 (2.39, 4.26)  Refrence  1.81 (1.34, 2.46) | <0.0001  0.0001 |
| RBC folate (ng/mL) | 464.00(351.00-605.00) | 1.001 (1.000, 1.001) | <0.0001 |
| Total cholesterol (mg/dl) | 191.18 ± 41.12 | 0.99 (0.99, 0.99) | <0.0001 |
| eGFR(mL/min/1.73m^2^)  White blood cells (10^3^ cells/ul)  Platelets (10^3^ cells/ul)  Albumin (g/dl)  Serum iron (ug/dl) | 97.22 ± 24.82  6.90(5.60-8.30)  236.81 ± 59.93  4.26 ± 0.34  80.00(60.00-104.00) | 0.99 (0.99, 0.99)  0.94 (0.91, 0.97)  1.005 (1.003, 1.006)  0.09 (0.07, 0.11)  0.97 (0.97, 0.97) | <0.0001  0.0002  <0.0001  <0.0001  <0.0001 |
| BMI (kg/m^2^) | 28.93 ± 6.98 | 1.02 (1.01, 1.03) | <0.0001 |
| Uric acid(mg/dl) | 5.42 ± 1.42 | 0.93 (0.88, 0.97) | 0.0013 |

Abbreviations: RBC red blood cells, BMI body mass index, eGFR estimated glomerular filtration rate
