# Supplementary material for: Association of serum uric acid with anemia in U.S. adults: a cross-sectional study using secondary data
Source: BMC Cardiovasc Disord. 2023 Jun 8;23:291. doi: 10.1186/s12872-023-03291-y (PMC10251553; doi:10.1186/s12872-023-03291-y)
Supplement: Supplementary file 2 — Additional File 2: Effect size of SUA on anemia in prespecified and exploratory subgroups [file 12872_2023_3291_MOESM2_ESM.doc]

**Supplemental table 2** Effect size of SUA on anemia in prespecified and exploratory subgroups

| Characteristic | No. of participants | OR (95%CI) | *P* for interaction |
| --- | --- | --- | --- |
| Age (year) |  |  | 0.7949 |
| <60 | 5803 | 1.03 (0.94, 1.14) |  |
| ≥60 | 2273 | 1.01 (0.91, 1.12) |  |
| Gender |  |  | 0.5527 |
| male | 4011 | 1.09 (0.97, 1.21) |  |
| female | 4065 | 1.04 (0.95, 1.14) |  |
| Race |  |  | 0.5015 |
| mexican American | 983 | 0.71 (0.54, 0.93) |  |
| non-Hispanic white | 3171 | 0.96 (0.84, 1.11) |  |
| non-Hispanic black | 1870 | 1.13 (1.01, 1.25) |  |
| other Hispanic | 804 | 0.87 (0.65, 1.16) |  |
| other races | 1248 | 0.92 (0.76, 1.12) |  |
| Educational level |  |  | 0.4696 |
| below high school | 1744 | 1.02 (0.89, 1.17) |  |
| high school | 1749 | 1.08 (0.94, 1.25) |  |
| above high school | 4583 | 0.97 (0.88, 1.07) |  |
| Smoking status |  |  | 0.3077 |
| never smoker | 4666 | 0.98 (0.89, 1.07) |  |
| former smoker | 1750 | 1.00 (0.87, 1.16) |  |
| current smoker | 1660 | 1.15 (0.96, 1.39) |  |
| Hypertension |  |  | 0.0378 |
| no | 5326 | 0.93 (0.84, 1.03) |  |
| yes | 2750 | 1.08 (0.98, 1.18) |  |
| Diabetes |  |  | 0.3237 |
| No | 7151 | 1.00 (0.92, 1.09) |  |
| Yes | 925 | 1.08 (0.95, 1.24) |  |
| eGFR |  |  | 0.3646 |
| ＜60 | 574 | 1.07 (0.92, 1.24) |  |
| ≥60 | 7502 | 0.99 (0.91, 1.07) |  |
| BMI |  |  | 0.6695 |
| ＜25 | 2453 | 1.02 (0.90, 1.17) |  |
| ≥25, ＜30 | 2645 | 1.04 (0.91, 1.19) |  |
| ≥30 | 2978 | 0.97 (0.87, 1.08) |  |

Note 1: Above model adjusted for age, gender, race, academic level, marital status, BMI, smoking status, drinking, RBC folate, Vitamin B12, total cholesterol, hypertension, diabetes mellitus, congestive heart failure, coronary heart disease, eGFR, white blood cells, platelets, albumin, serum iron.

Note 2: In each case, the model is not adjusted for the stratification variable
